# A new species of the genus *Hydrodroma* Koch, 1837 (Acari, Hydrachnidia, Hydrodromidae), with a key to the hitherto known six species of the genus in Australia

**DOI:** 10.3897/zookeys.143.2115

**Published:** 2011-11-01

**Authors:** Vladimir Pešić, Harry Smit

**Affiliations:** 1 Department of Biology, University of Montenegro, Cetinjski put b.b., 81000 Podgorica, Montenegro; 2 Netherlands Centre for Biodiversity Naturalis, P.O. Box 9517, 2300 RA Leiden, The Netherlands

**Keywords:** water mites, new species, taxonomy

## Abstract

The genus *Hydrodroma* Koch, 1837 in Australia consists of six species, the newly described *Hydrodroma meridionalis* **sp. n.** included. The new species is described from 45 sampling sites from running waters in Queensland, Victoria, New Southern Wales, Western Australia, Northern Territory and South Australia. Furthermore, a key for the identification of species of *Hydrodroma* occurring in Australia is given.

## Introduction

The genus *Hydrodroma* Koch, 1837has been found in all biogeographic regions except Antarctica. However, the taxonomy and systematics of the genus is difficult (Pešić and Smit 2007a, b). The adult and nymphal stages are characterized primarily by the number and distribution of swimming setae, body colour, morphology and chaetotaxy of the genital field and idiosoma structure (Wiles 1985, Di Sabatino et al. 2010).

Recently, Pešić and Smit (2007a, b) showed that Australian populations of the genus *Hydrodroma*, formerly reported as *Hydrodroma despiciens* (Müller, 1776), *Hydrodroma monticola* (Piersig, 1906), *Hydrodroma* sp. A *sensu* Cook, 1986 and *Hydrodroma* sp. B *sensu* Cook, 1986 (see: Lundblad 1947, Szalay 1953, Cook 1986 and Harvey 1998) represent several clearly distinct species. Thus far, five species have been described from Australia (Pešić and Smit 2007a, b), i.e. *Hydrodroma kununurra* Pešić & Smit, 2007, *Hydrodroma australis* Pešić & Smit, 2007, *Hydrodroma kakadu* Pešić & Smit, 2007, *Hydrodroma wilesi* Pešić & Smit, 2007 and *Hydrodroma cooki* Pešić & Smit, 2007.

This paper gives the description of a new species and a key for the identification of *Hydrodroma* species occurring in Australia.

## Materials and methods

Water mites were collected by hand netting, sorted on the spot from the living material, preserved in Koenike-fluid and dissected as described by Davids *et al*. (2007). The holotype and some of the paratypes will be deposited in Queensland Museum in Brisbane (QM), other paratypes and non-type material in the Netherlands Centre for Biodiversity Naturalis in Leiden (RMNH). Unless stated otherwise, all material has been collected by the junior author and this is not repeated in the text.

The composition of the material is given as: males/females/deutonymphs or adults/deutonymphs. All measurements are given in micrometers (µm). The following abbreviations are used: Cx-I = first coxae, dL = dorsal length, H = height, L = length, I/II/III/IV-Leg-1-6 = first to sixth segments of leg I/II/III/IV, IV-Leg-5a = anterior surface of leg IV, segment 5; IV-Leg-5p = posterior surface of leg IV, segment 5; P-1 to P-5 = palp segments 1 to 5, *x¯*= mean values, n = number of specimens examined, NP = National Park, vL = ventral length, W = width.

## Systematics

### Hydrodromidae K. Viets, 1936. Genus Hydrodroma Koch, 1837

#### Hydrodroma meridionalis sp. n.

urn:lsid:zoobank.org:act:07CBCA67-AB0B-4191-A7E2-15744527AAE2

http://species-id.net/wiki/Hydrodroma_meridionalis

**Figure 1.**
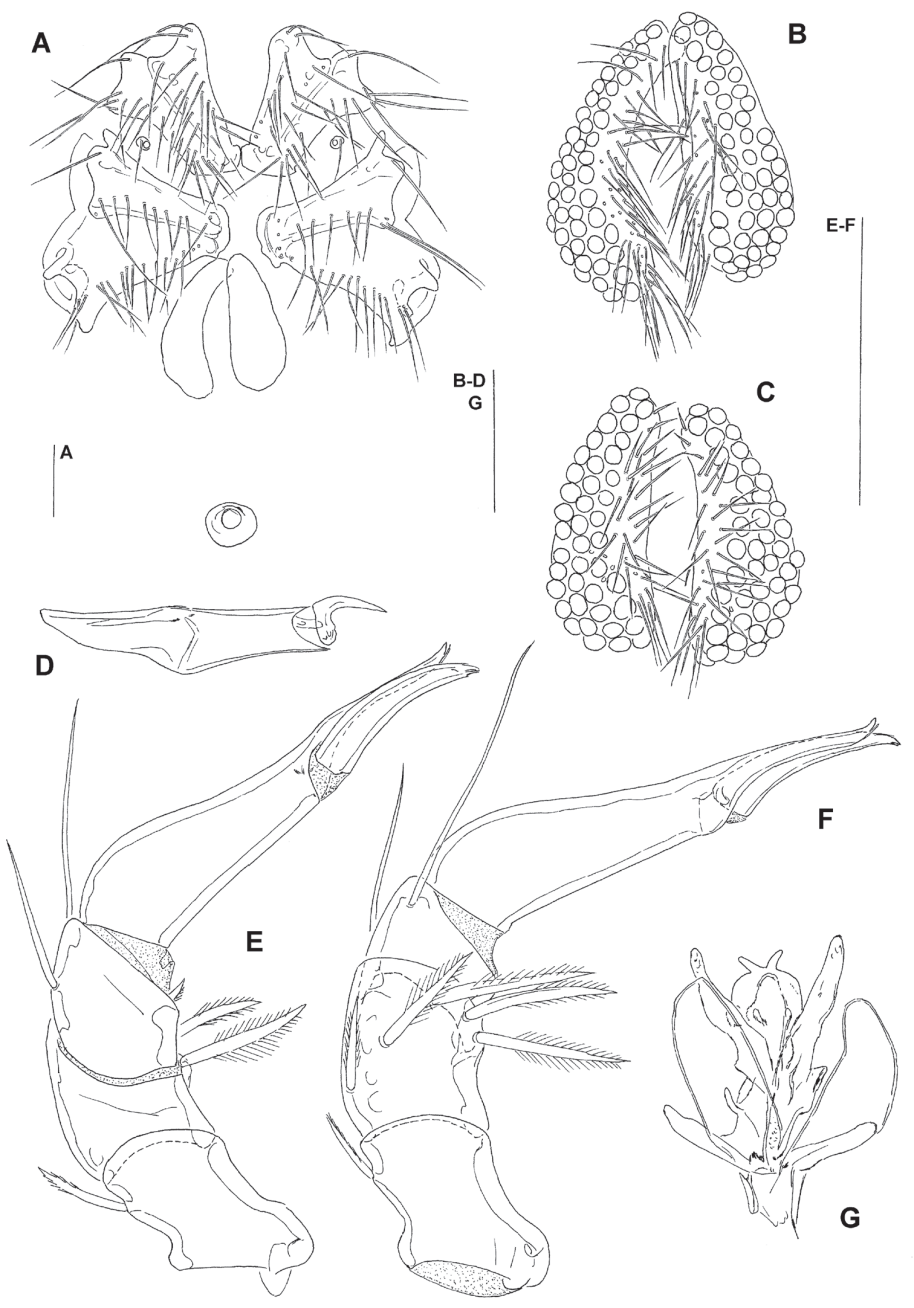
** A–G** *Hydrodroma meridionalis* sp.n. (**A–B, D, G** = male holotype, **C, E, F** = female paratype) **A** = coxal and genital field **B–C** = genital field **D** = chelicera **E** = palp, lateral view **F** = palp, medial view **G** = ejaculatory complex. Scale Bars = 100 μm.

Figs 1A–G 2B 3A Tables 1 [Table T2] 3 

##### Type series.

 Holotype male, dissected and slide-mounted, Queensland, Lawn Hill Creek, cascades, Lawn Hill NP, 10.v.2005, 18°41.806S, 138°29.138E (QM). Paratypes: 12 males, 8 females, same data as holotype, one male and one female of them dissected and slide-mounted in Hoyer's fluid (QM); 35/33/0, Lawn Hill Creek at campground, Lawn Hill NP, 10.v.2005, 18°42.011S, 138°29.235E, four males and two females of them dissected and slide-mounted in Hoyer's fluid (RMNH).

##### Further records.

 QUEENSLAND: Nankin Creek, Rockhampton, 04.v.1981, leg. A.P. Mackey, 2/3/0; ibid., 20.vii.1981, 2 [damaged]/2 [damaged]/0; ibid., 06.ii.1982, 1/1 [damaged] /0; Innot Hot Springs, 11.viii.1989, 1/0/0; Broken River near Conical Pool, Eungella NP, 18.ix.2000, 1/2/0; Crediton Creek, Eungella NP, 18.ix.2000, 2/7/0; The Millstream, upstream of Millstream Falls, Millstream NP, 16.ix.2000, 0/10/0; Wenlock River at crossing with road to Iron Range NP, 06.ix.2000, 1/1/0; Little Yabba Creek, S of Kenilworth, 20.ix.2000, 2/9/0; Cattle Creek at crossing with road to Finch Hatton Gorge, W of Mackay, 19.ix.2000, 0/6/1; Fletcher Creek, Dalrymple NP, 22.x.2005, 19°49.125S, 146°03.771E, 6/7/0; Alligator Creek, Bowling Green Bay NP, 22.x.2005, 19°26.192S, 146°56.862E, alt. 32 m a.s.l., 5/10/0 (0/1/0 mounted); Gregory River at Gregory Downs, 11.x.2005, 8°38.811S; 139°15.008E, alt. 68 m a.s.l., 30/0; Waterview Creek at Jourama Falls, Paluma Range NP, 20.x.2005, 18°51.729S, 146°07.650E, 1/0/0; Davies Creek, Davies Creek NP, 13.x.2005, 17°00.212S, 145°34.180E, 1/2/0; Little Yabba creek at Charlie Moreland Campground, Kenilworth, 02.xi.2005, 26°36.928S, 152°39.105E, 11/15/0. NEW SOUTH WALES: School Creek near Morton NP, 05.xi.2001, 0/1/0; Upper Kangaroo River, N of Kangaroo Valley, 07.xi.2001, 1/1/0; Wattamolla Creek, Royal NP, 08.xi.2001, 0/2/0; tributary of Sawyers Creek, S of Kangaroo Valley, 06.xi.2001, 2/2/0; Bugong Creek near border of Morton NP, 05.xi.2001, 6/9/1; Nymboida River at Platypus Flat, Nymboi-Binderay NP, 09.xi.2003, 30°11.146S, 152°41.499E, alt. 443 m a.s.l., 2/1/0; Urumbilum River, Bindarri NP, 7.xi.2003, 30°15.966S, 152°57.042E, alt. 137 m a.s.l., 3/2/0 (1/0/0 mounted); Mann River at Mann River Nature Reserve, 20.xi.2003, 29°41.291S, 152°05.815E, alt. 403 m a.s.l., 2/2/0 (1/0/0 mounted); Towamba River at Big Jack Rest Area, South East Forests NP, 11.xii.2003, 31°53.885S, 149°27.807E, alt. 271 m a.s.l, 1/6/0; Wog Wog River at crossing with Wog Way, 10.xii.2003, 37°04.986S, 149°29.027E, alt. 332 m a.s.l., 1/0/2; Bellinger River at Gordanville Crossing, 22.xi.2003, 30°25.067S, 152°50.845E, alt. 20 m a.s.l., 1/1/0; Minnemurra River at Minnemurra Rainforest, 18.xii.2003, 34°38.183S, 150°43.272E, 0/2/2; NORTHERN TERRITORY: Douglas River at Douglas Hot Springs, 01.viii.1994, 13°46S, 131°26E, 3/1/0 (1/1/0 mounted); Pond Chinaman Creek, 16 km S of Katherine, 29.vii.1994, 7/7/1; Pool near Jim Jim Falls, Kakadu NP, 23.vii.1994, 2/0/0; Katherine River near Visitors Centre, Katherine Gorge NP, 28.vii.1994, 4/1/0; 17 Mile Creek, tributary of Katherine River, Katherine Gorge NP, 28.vii.1994, 0/2/0. WESTERN AUSTRALIA: Pool Lennard River, Windjana Gorge NP, 09.ix.1998, 1/1/0; Plunge pool, The Grotto, S of Wyndham, 20.ix.1998 1/0/0; Pool Lennard Gorge, The Kimberley, 1.ix.1998, 2/1/0; Pool Valentine Springs, W of Kununurra, 18.ix.1998, 0/2/0; Plunge Pool Black Rock Falls, W of Kununurra, 18.ix.1998, 0/1/0; pools 3 km W of Lennard Gorge, The Kimberley, 10.ix.1998, 1/1/0; unnamed creek at crossing with Windjana Gorge road, 38 km N of Great Northern Highway, 30.ix.1998, 2/9/0; pool west of Tunnel Creek, Tunnel Creek NP, 30.ix.1998, 6/6/0. VICTORIA: Crystal Brooke at Hospice Plain, Mt Buffalo NP, 10.x.1997, 0/1/0; Stony Creek downstream of Turret Falls, Grampians NP, 17.iii.2008, 37°09.662S, 142°29.789E, alt. 517 m a.s.l., 0/1/0; Mt Williams Creek, downstream of Kalymna Falls, Grampians NP, 18.iii.2008, 37°19.034S, 142°36.212E, 1/0/0; Rockpool Buandik Falls, 16.iii.2008, 37°14.803S, 142°16.914E, 1/2/0; Stringers Creek upstream of Walhalla, 09.iii.2008, 37°56.006S, 146°26.926E, alt. 360 m a.s.l., 0/1/0; Jump Creek, Mt Buffalo NP, 11.iii.2008, 36°46.350S, 146°47.636E, alt. 1468 m a.s.l., 1/1/0;. SOUTH AUSTRALIA: Onkaparinga River at Sundews Trail, Onkaparinga NP., 06.iv.2008, 35°09.478S, 138°34.791E, alt. 95 m a.s.l., 5/3/0.

##### Diagnosis.

 Genital plate with 37–52 acetabula in 3–4 longitudinal rows; palp segments narrow (L/H ratio P-4 4.6–5.2, in both sexes); number of swimming setae: II-Leg-5 1; III-Leg-4p 7–9, III-Leg-5p 5–7, IV-Leg-4a 6–12, IV-Leg-4p 6–12, IV-Leg-5a 2–4, IV-Leg-5p 2–8.

##### Description.

**FIgure 2.**
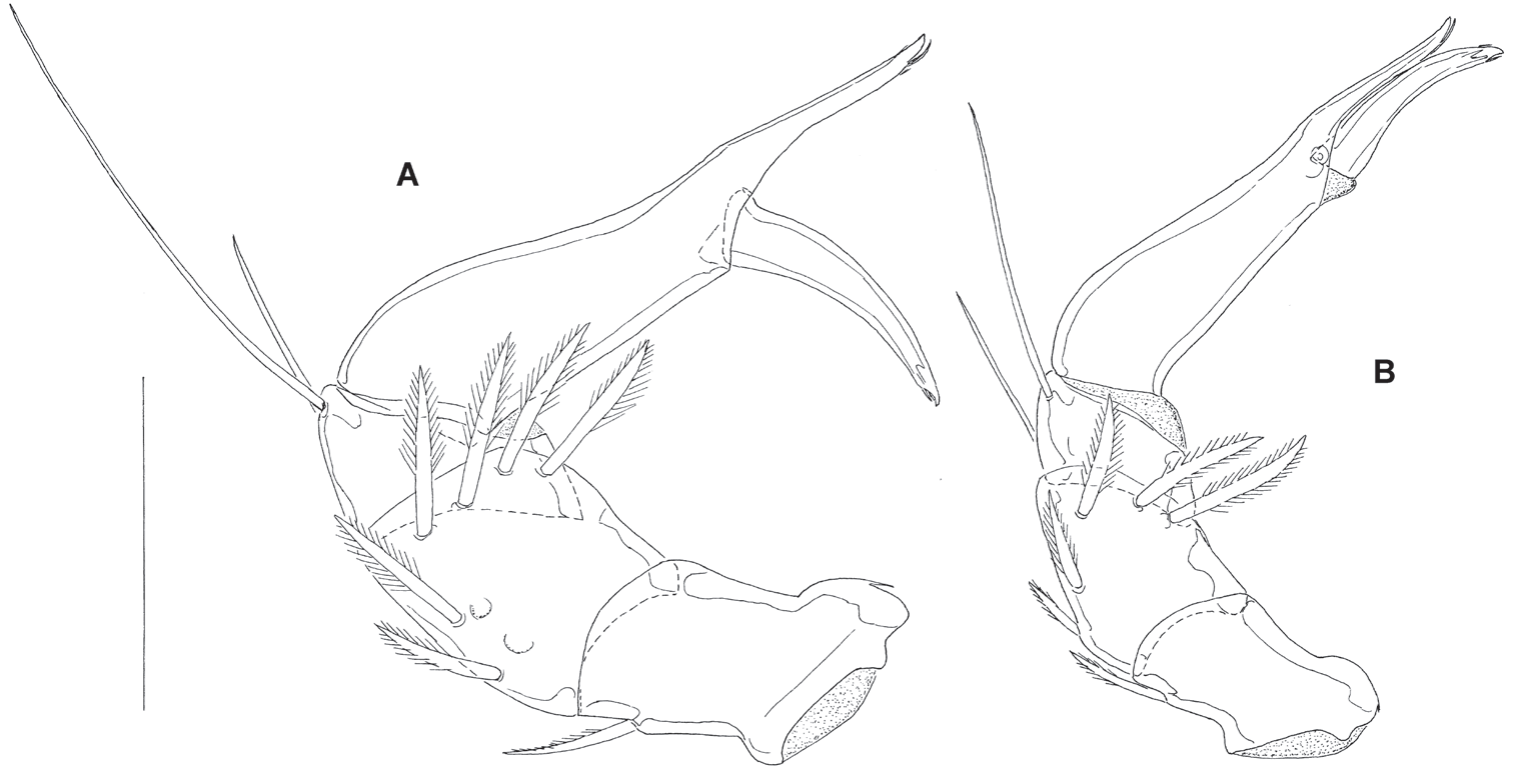
**A** *Hydrodroma torrenticola* (Walter, 1908), male (Croatia, Ombla spring): palp, medial view **B** *Hydrodroma meridionalis* sp. n., male holotype: palp, medial view. Scale bar = 100 μm.

Male. (holotype; in parentheses measurements of paratypes, if not given otherwise n = 5): Idiosoma L/W (800–994/680–813); integument papillae bluntly pointed. Coxal field: L Cx-I+II, 208 (206–226, *x¯* = 215), Cx-III+IV, 244 (241–263, *x¯*  = 252), total number of coxal setae: 15–18 (18–22, *x¯* = 20) on Cx-I, 18–19 (16–21, *x¯* = 18) on Cx-II, 14 (11–16, *x¯* = 14) on Cx-III, 17 (15–19, *x¯* = 17) on Cx-IV. Genital plate (Fig. 1B, 3A): setae more numerous than in females, for measurements, Ac and setae numbers see Table 1; ejaculatory complex L 163 (163–177, n = 3, *x¯* = 169). Capitulum vL 183 (188–203, *x¯* = 195); chelicera (Fig. 1D) total L 247 (265–275, *x¯* = 270), basal segment L 194 (209–216, *x¯* = 213), claw L 51 (52–55, *x¯* = 54). Palp as in female, for chaetotaxy see Fig. 2B, for measurements see Table 1. Number of swimming setae on legs are presented in Table 2.

Female. (paratypes, n = 3): Idiosoma L/W 840–1044/750–938. Coxal field: L Cx-I+II, 219, Cx-III+IV, 247; number of coxal setae: 18–23 (*x¯* = 21) on Cx-I, 14–23 (*x¯* = 19) on Cx-II, 12–16 (*x¯* = 14) on Cx-III, 14–19 (*x¯* = 17) on Cx-IV. Shape of genital plate as in Fig. 1C, for measurements, Ac and setae numbers see Table 1. Capitulum vL 203–225 (*x¯* = 217); chelicera total L 269–286 (*x¯* = 278), basal segment L 214–235 (*x¯* = 224), claw L 54–60 (*x¯* = 56). Palp: For chaetotaxy see Fig. 1F, for measurements see Table 1. Numbers of swimming setae on legs are presented in Table 2.

**Figure 3.**
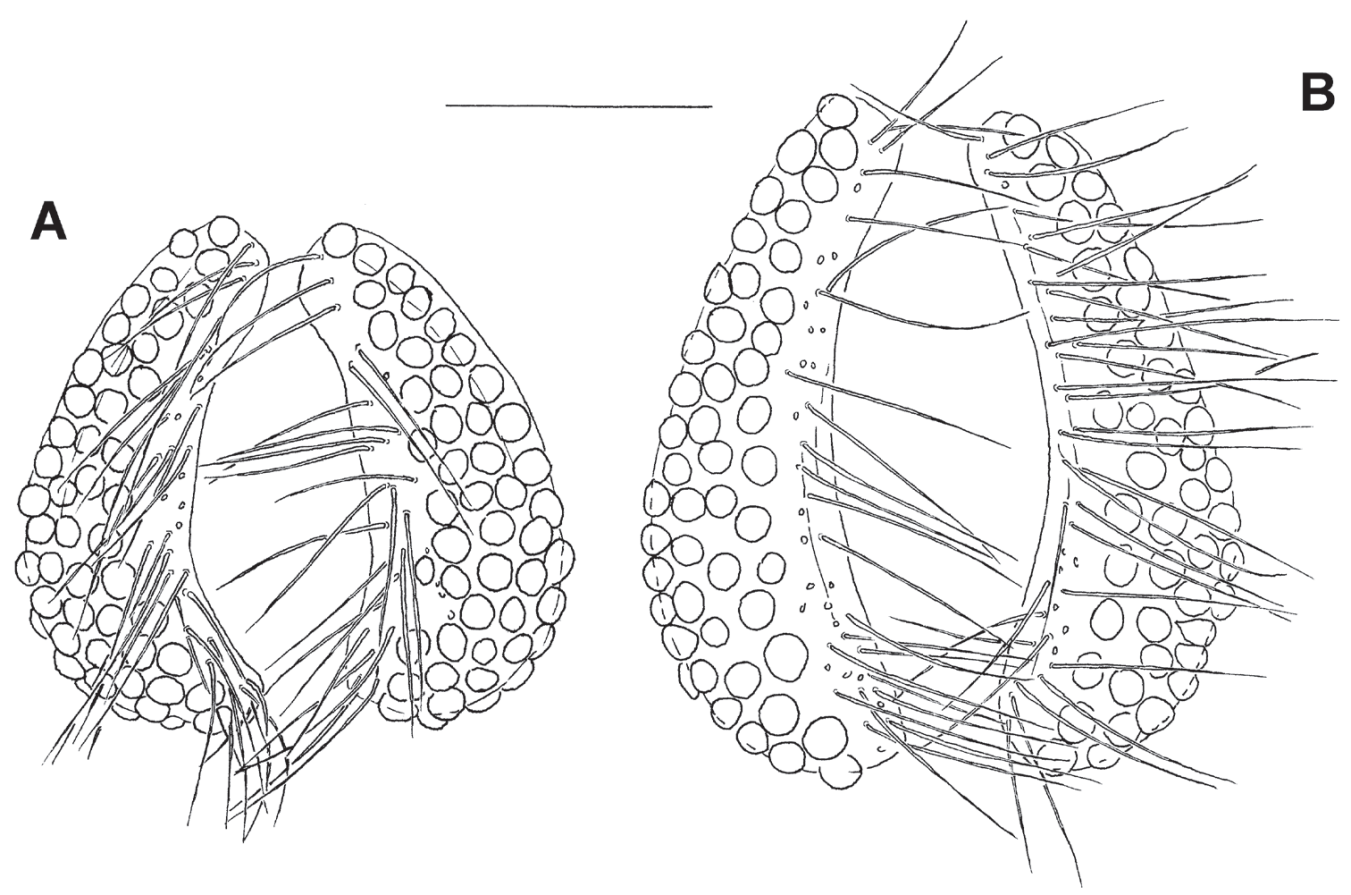
**A** *Hydrodroma meridionalis* sp. n., male paratype: genital field **B** *Hydrodroma torrenticola* (Walter, 1908), male (Serbia, Kozjak Mt., stream): genital field. Scale bar = 100 μm.

**Table 1.** Morphometric data for the genital plate, palp and leg segments (2–6) for *Hydrodroma meridionalis* sp. n. Numbers (n) and length in µm (L) are given.

	MALE		FEMALE
	holotype	paratype (n =5, in parentheses *x¯*)	paratype (n =3)
genital acetabula, n	47–46	42–51 (49)	37–52 (45)
genital setae, n	32–46	35–43 (38)	27–33 (31)
genital plate, L	191–195	184–200 (192)	175–195 (185 )
dL P-1	39	43–47 (45)	46–63 (53)
dL P-2	65	63–65 (65)	64–70 (66 )
dL P-3	44	35–48(42)	43–52 (48)
dL P-4	158	163–168 (165)	168–182 (176)
dL P-5	67	62–67 (66)	69–72 (70)
Palp, total L	373	376–390 (383)	390–428 (413)
H P-4	32	32–34 (33)	35–39 (36)
L/H P-4 ratio	4.9	4.8–5.1 (5.0)	4.6–5.2 (4.9)
dL I-Leg-2	68	72–75 (74)	73–86 (80)
dL I-Leg-3	94	97–100 (99)	95–109 (103)
dL I-Leg-4	141	143–147 (145)	144–172 (157)
dL I-Leg-5	184	184–194 (189)	194–222 (207)
dL I-Leg-6	172	166–184 (179)	178–194 (189)
dL II-Leg-2	88	91–97 (93)	97–103 (100)
dL II-Leg-3	116	116–122 (118)	119–137 (128)
dL II-Leg-4	191	197–203 (201)	203–234 (220)
dL II-Leg-5	234	238–250 (245)	244–281 (261)
dL II-Leg-6	203	200–220 (214)	191–226 (214)
dL III-Leg-2	91	94–103 (98)	100–116 (109)
dL III-Leg-3	109	111–119 (116)	115–131 (125)
dL III-Leg-4	176	185–191 (189)	192–222 (208)
dL III-Leg-5	219	222–234 (229)	234–267 (249)
dL III-Leg-6	198	191–217 (208)	210–229 (220)
dL IV-Leg-2	122	128–141 (132)	134–159 (146)
dL IV-Leg-3	159	159–178 (169)	175–200 (187)
dL IV-Leg-4	244	244–259 (252)	259–294 (276)
dL IV-Leg-5	261	259–281 (272)	281–322 (299)
dL IV-Leg-6	239	231–252 (245)	255–275 (266)

**Table T2:** Table 2. Number of swimming setae of *Hydrodroma meridionalis* sp. n., from Lawn Hill Creek (type series).

	*Hydrodroma meridionalis* sp. n.	
	male holotype (paratype, n =7)	female (n = 5)
II-Leg-5p	1 (1)	1
III-Leg-4p	8 (8–9)	7–9
III-Leg-5p	6 (5–7)	5–7
IV-Leg-4a	8–9 (8–10)	9–10
IV-Leg-4p	8–9 (8–9 )	8–10
IV-Leg-5a	2–3 (2–3)	1–3
IV-Leg-5p	4–5 (4–6)	3–6

**Table 3.** Number of swimming setae of *Hydrodroma meridionalis* sp. n. (Victoria: Stony Creek 1♀, Rockpool Buandik Falls 2♀, Jump Creek 1♂; Queensland: Gregory River 2♂, 2♀, Fletcher Creek 3♀, Cattle Creek 1♀, Waterview Creek 1♂, Davies Creek 1♂, 1♀, Alligator Creek 1♀, Broken River 2♀; New South Wales: Kangaroo Valley 1♂, 1♀, Bugong Creek 2♂, 1♀, Towamba River 1♀, Wog Wog River 1♂; Western Australia: Plunge pool, The Grotto, S of Wyndham, 1♂, Pool Lennard River 2♀,1♂, S of Kununnura 2♀, pool W of Tunnel Creek 2♂,1♀, unnamed creek at crossing with Windjana Gorge road 1♀; Northern Territory: Douglas River 1♂, 1♀, Chinaman Creek 2♂, 3♀).

	Victoria		Queensland		New Southern Wales		Western Australia		Northern Territory	
	male (n =1)	female (n =3)	male (n =4)	female (n =10)	male (n =4)	female (n =3)	male (n =4)	female (n =7)	male (n = 3)	female (n = 4)
II-Leg-5p	1	1	1	1	1	1	1	1	1	1
III-Leg-4p	8	9–12	8–9	8–9	8–12	10–12	7–11	6–9	8–9	7–12
III-Leg-5p	6	6–9	6–5	4–6	7–10	8–11	3–6	3–7	5–6	5–8
IV-Leg-4a	8	8–12	7–10	6–11	7–10	9–11	7–9	6–9	7–8	7–9
IV-Leg-4p	8	9–11	6–9	7–11	7–11	10–12	7–10	7–10	7–9	7–10
IV-Leg-5a	2	3–4	2–3	1–3	2–3	3–4	0–2	1–2	2–3	0–2
IV-Leg-5p	5–6	7–8	3–5	3–5	4–8	5–6	4	3–4	4–5	2–6

##### Remarks.

*Hydrodroma meridionalis* sp. n. is most similar to the European *Hydrodroma torrenticola* (Walter, 1908), in the presence of one swimming seta on II-Leg-5, IV-Leg-5 anteriorly with 2–5 swimming setae and the presence of relatively large-sized leg claws.

*Hydrodroma torrenticola* (in parentheses data combined from Wiles 1986, Di Sabatino et al. 2010 and our material from Croatia and Serbia) differs from *Hydrodroma meridionalis* sp. n., in larger dimensions of the genital plates in the both sexes (L 225–275 µm), a more slender genital plate in the male (compare Figs 3A with 3B), a longer ejaculatory complex (L > 200 µm), stouter palp segments, especially P-4 (compare Figs 2A with Fig. 2B) and generally a lower number of swimming setae on III-Leg-4 (5–8 swimming setae).

##### Variability.

 We found variability in the number of swimming setae (Table 3). The populations from Western Australia are characterized by generally lower number of swimming setae on anterior IV-Leg-5 (1–2 swimming setae, occasionally setae reduced on one side).

##### Etymology.

 Named for its southern occurrence.

##### Habitat.

 Most specimens were taken from pools of low order streams or from lotic areas of slow flowing streams. Like *Hydrodroma torrenticola*, the new species is obviously rheophilous.

##### Distribution.

 Widespread in Australia (Queensland, Victoria, New Southern Wales, Western Australia, Northern Territory, South Australia).

### Key to the Australian species of Hydrodroma Koch, 1837

**Table:** 

1	II-Leg-5 with more than four swimming setae	2
–	II-Leg-5 with one or without swimming setae	4
2	IV-Leg-5 anteriorly without swimming setae	*Hydrodroma kakadu* Pešić & Smit, 2007additional characters: genital plates with 28–47 Ac in 3–4 rows, number of swimming setae: II-Leg-5p 3–8, III-Leg-4 8–10, III-Leg,5 5–9, IV-Leg-4a, 7–9 IV-Leg-4p 7–9, IV-Leg-5p 3–6
–	IV-Leg-5 anteriorly with 2–5 swimming setae; number of Ac and swimming setae various	.3
3	Genital plate with < 50 Ac in 3–4 rows	*Hydrodroma australis* Pešić & Smit, 2007additional characters: ejaculatory complex L < 210, number of swimming setae: II-Leg-5p 4–6, III-Leg-4 9–14, III-Leg-5 7–10, IV-Leg-4a 7–12, IV-Leg-4p 7–12, IV-Leg-5a 2–4, IV-Leg-5p 4–8
–	Genital plate > 70 Ac in 5–6 rows	*Hydrodroma kununurra* Pešić & Smit, 2007additional characters: ejaculatory complex L > 210, number of swimming setae: II-Leg-5p 6–9, III-Leg-4 12–19, III-Leg-5 10–12, IV-Leg-4a 11–14, IV-Leg-4p 13–18, IV-Leg-5a 4–6, IV-Leg-5p 9–12
4	IV-Leg-5 anteriorly with 2–4 swimming setae (usually with two swimming setae, occasionally with a single seta, or setae are reduced on one side; the number of setae should be checked in more specimens)	*Hydrodroma meridionalis* sp. n.additional characters: genital plates with 37–52 Ac in 3–5 rows, number of swimming setae: III-Leg-4p 7–9, III-Leg-5p 5–7, IV-Leg-4a 6–12, IV-Leg-4p 6–12, IV-Leg-5p 2–8
–	IV-Leg-5 anteriorly without swimming setae, number of Ac and swimming setae various	5
5	Genital plate with < 60 Ac in 4–5 rows; legs with a relatively large-sized claw	*Hydrodroma wilesi* Pešić & Smit, 2007additional characters: number number of swimming setae: III-Leg-4 1–2, III-Leg-5 1–3 rather short, IV-Leg-4a 2–3, IV-Leg-4p 2–4, IV-Leg-5p 1
–	Genital plate with > 110 Ac in 5–9 rows; legs with a relatively small-sized claw	*Hydrodroma cooki* Pešić & Smit, 2007additional characters: number of swimming setae: III-Leg-4 > 10, III-Leg-5 7–13, IV-Leg-4a 8–14, IV-Leg-4p 10–16, IV-Leg-5p 6–11

## Supplementary Material

XML Treatment for Hydrodroma meridionalis
